# Supplementary figures and images for: Parameterization of intraoperative human microelectrode recordings: Linking action potential morphology to brain anatomy
Source: PLoS Comput Biol. 2025 Jun 17;21(6):e1013184. doi: 10.1371/journal.pcbi.1013184 (PMC12204630; doi:10.1371/journal.pcbi.1013184)

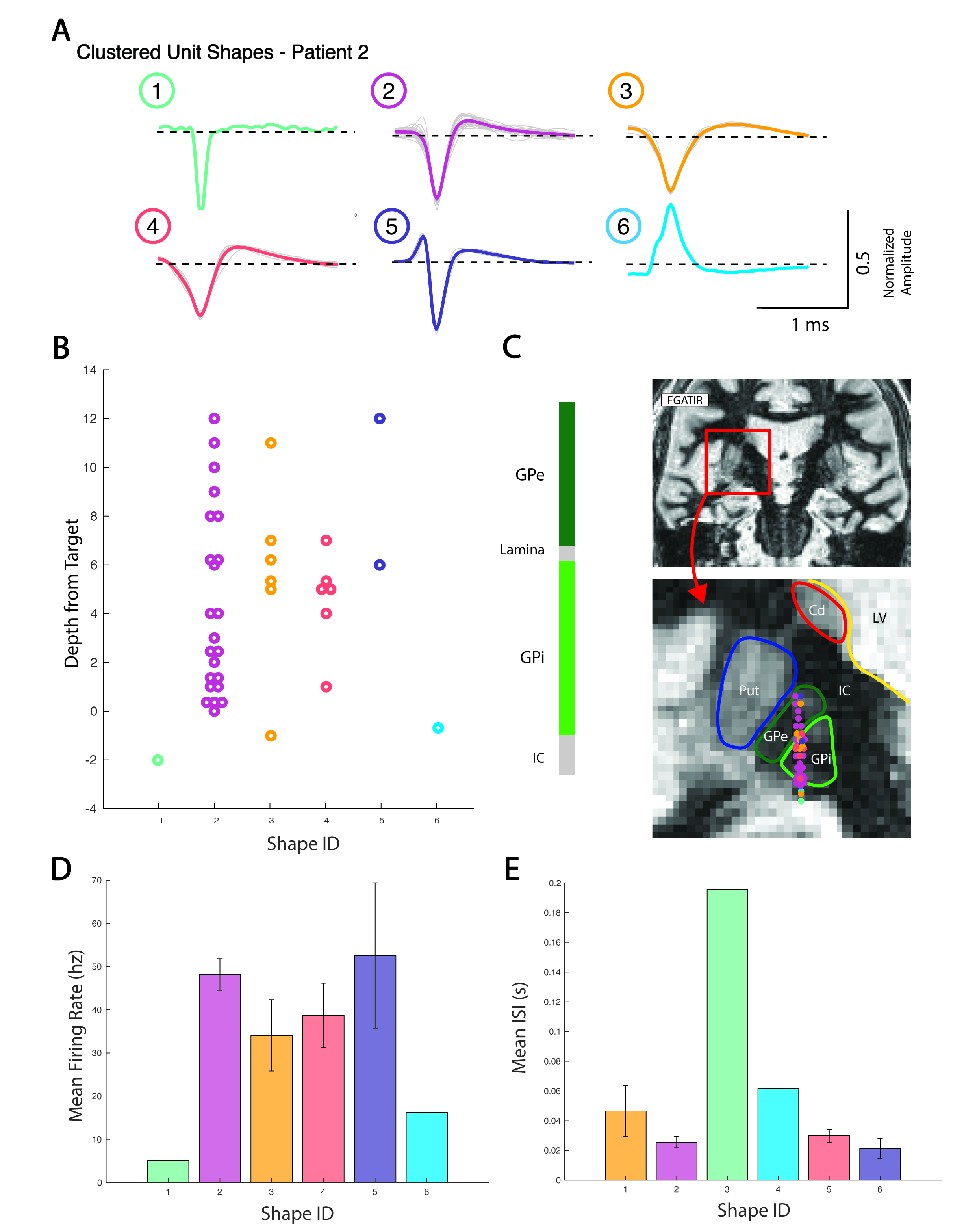

Supplement: S1 Fig — (A) Normalized unit waveforms and average cluster shape for the 6 unique shapes identified from hierarchical clustering. Shapes 2, 3, and 4 are the most common shapes, consistent with figures 7-8. (B) The 6 clustered spike shapes plotted by depth from target (mm) in the internal globus pallidus (GPi). (C) Spike shapes are plotted along the visualized microelectrode recording track on hand-segmented anatomy on psuedocoronal FGATIR MRI. Average +/- SEM firing rate (D) and interspike interval (E) across shape clusters. (TIF) [file pcbi.1013184.s001.tif]

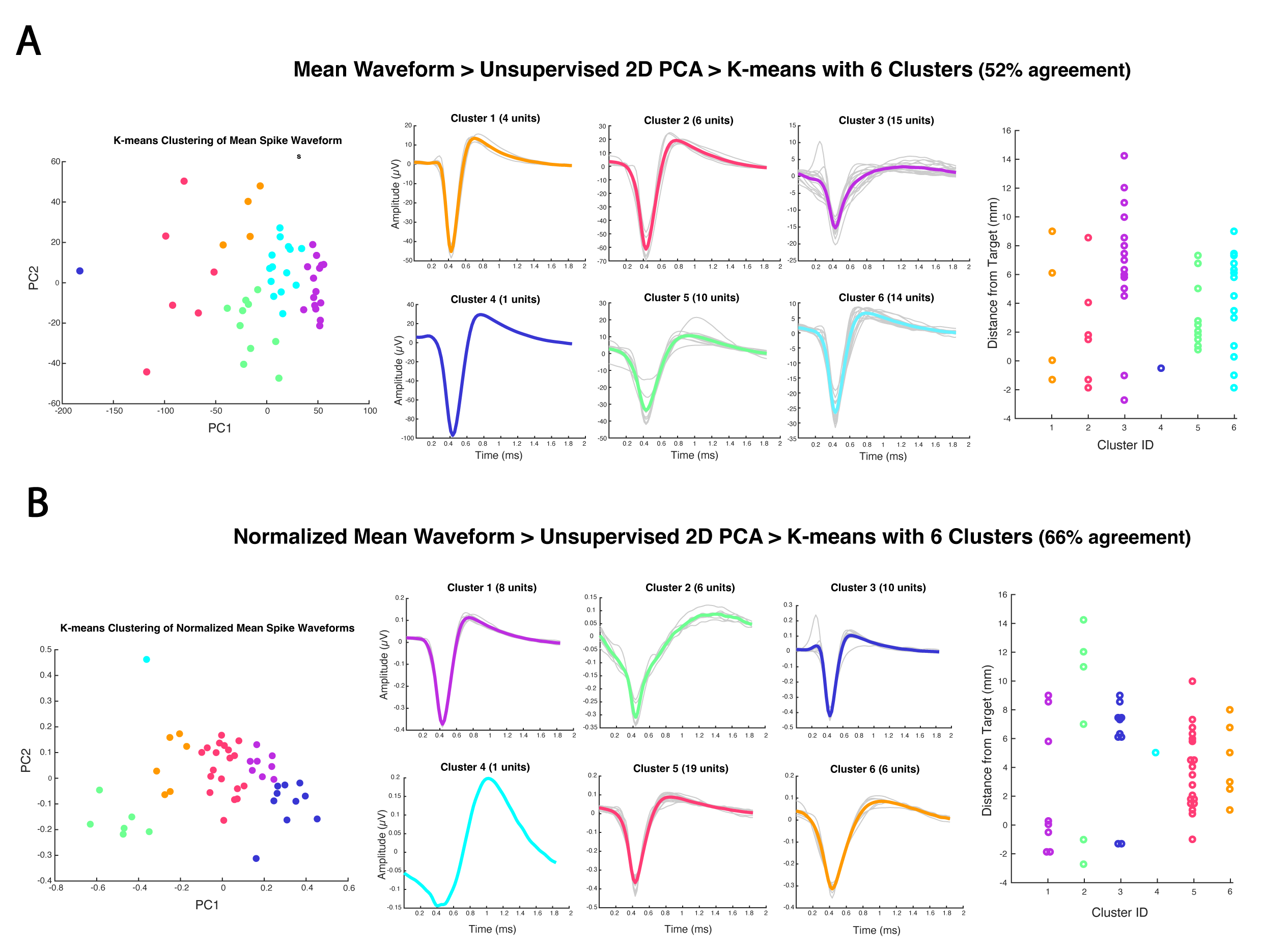

Supplement: S2 Fig — We compared hierarchical clustering outcomes from Figs 7 and 8 to directly clustering spike-sorted mean waveforms through unsupervised PCA (2D) followed by K-means clustering (specified same number of clusters) and compared overlap using the Hungarian method, a clustering optimization algorithm. (A) Directly clustering mean unit waveforms shared 52% overlap with the hierarchical clustering method, and were heavily influenced by unit amplitude. (B) By first normalizing the mean unit waveforms prior to PCA and clustering, the spike shapes more closely resembled our clustering findings, but still only shared 66% overlap. (TIF) [file pcbi.1013184.s002.tif]
