# Supplementary material for: Parameterization of intraoperative human microelectrode recordings: Linking action potential morphology to brain anatomy
Source: PLoS Comput Biol. 2025 Jun 17;21(6):e1013184. doi: 10.1371/journal.pcbi.1013184 (PMC12204630; doi:10.1371/journal.pcbi.1013184)
Supplement: Attachment — Submitted filename: reviewer response.pdf [file pcbi.1013184.s003.pdf]

MAYO CLINIC

April 28th, 2025

Dear Editors of PLOS Computational Biology,

This is a reply to Reviewers for the manuscript "Parameterization of intraoperative human microelectrode recordings: Linking action potential morphology to brain anatomy" (PCOMPBIOL-D-25-00122R1) by Baker, Klassen, Jensen, Valencia, Heydari, Ince, Müller, and Miller.

We would like to thank the Reviewers for their very thoughtful feedback and interest in our manuscript. We have added additional text, analyses, and figure panels to address the Reviewers' concerns. We believe that these revisions significantly improve the manuscript and serve to further underscore our important findings - we are very grateful for the Reviewers' suggestions.

In our point-by-point reply below, gray text indicates commentary from the Reviewer, **black text** indicates author response, and blue text indicates quotations from the manuscript.

We again thank the Editors for their consideration and the Reviewers for their time and helpful feedback. We are proud that our work is being considered for publication at PLOS Computational Biology.

Sincerely yours,

Matthew R Baker, Mayo Clinic Department of Neurosurgery, on behalf of all authors
Baker.matthew@mayo.edu

Reviewer's Responses to Questions

Reviewer #1: This paper presents a novel algorithmic approach to parameterize intraoperative human microelectrode recordings (MERs) to improve deep brain stimulation (DBS) targeting. The method uses canonical response parameterization (CRP) to characterize the structure and duration of action potentials, allowing for the clustering of spike morphologies based on their similarity. By applying this technique to DBS recordings, the authors found that spike shapes clustered regionally in the human globus pallidus, suggesting anatomical correlations. This approach enhances the precision of DBS lead placement and holds potential for broader applications in neurosurgical procedures.

This is a wonderful paper, with a refreshing take on spike analysis, following their already impressive work on CCEPs. However, there are a few areas where the manuscript could be improved.

1. The dataset used is small, consisting of only 3 patients. This is surprising, given that Mayo is such a clinically busy hospital. Including more patients, or using any of the various open-access MER datasets available on DABI or elsewhere, would help ensure the generalizability of this method.

We agree with the reviewer that the small cohort makes it difficult to make definitive statements and generalize our findings. However, the purpose of this study is to introduce a new methodological framework to parameterize and compare morphological features of sorted spike data, and present several different contexts in which it might be useful. We have added spike shape clustering for the third patient (Figure S1), which shows the same 3 common shapes of sorted units across similar spans – but also emphasize in our new limitations section that future work is ongoing to apply this to large group level data to identify broader patterns in spike shape, firing, and anatomy.

We have added the following limitations section at the bottom of the results/discussion:

"There are important limitations to consider with this approach. First, this study was conducted on densely sampled recordings from a small number of patients, which limits the generalizability of our findings. Future studies will assess group-level clustering and create classifier models to predict clinically-labeled boundaries from spike shapes and firing. While we observed distinct spike shapes across anatomical regions that were largely consistent in both patients, other factors beyond cell-type such as cellular orientation, glial interactions, and neuromodulatory tone may influence waveform structure. While our method normalizes unit amplitude to reduce the impact of electrode distance, any ground truth assessment of cell-type would require a mixture of transgenic animal models, optogenetics, histology, and others. Additionally, some spike shapes observed in white matter regions may reflect some combination of background activity and amplifier/background noise rather than discrete units and should be interpreted cautiously. Regardless, even reliably identifiable shapes from background spiking/noise could still prove useful for classifier models. While this approach offers advantages over existing techniques, such as empirical spike windowing and shape based similarity, it is designed to complement, not replace, standard spike sorting and shape comparison methods. Although, principles could be integrated into automated pipelines to enhance spike detection and unit classification similar to other matching pursuit methods."

Figure S1:

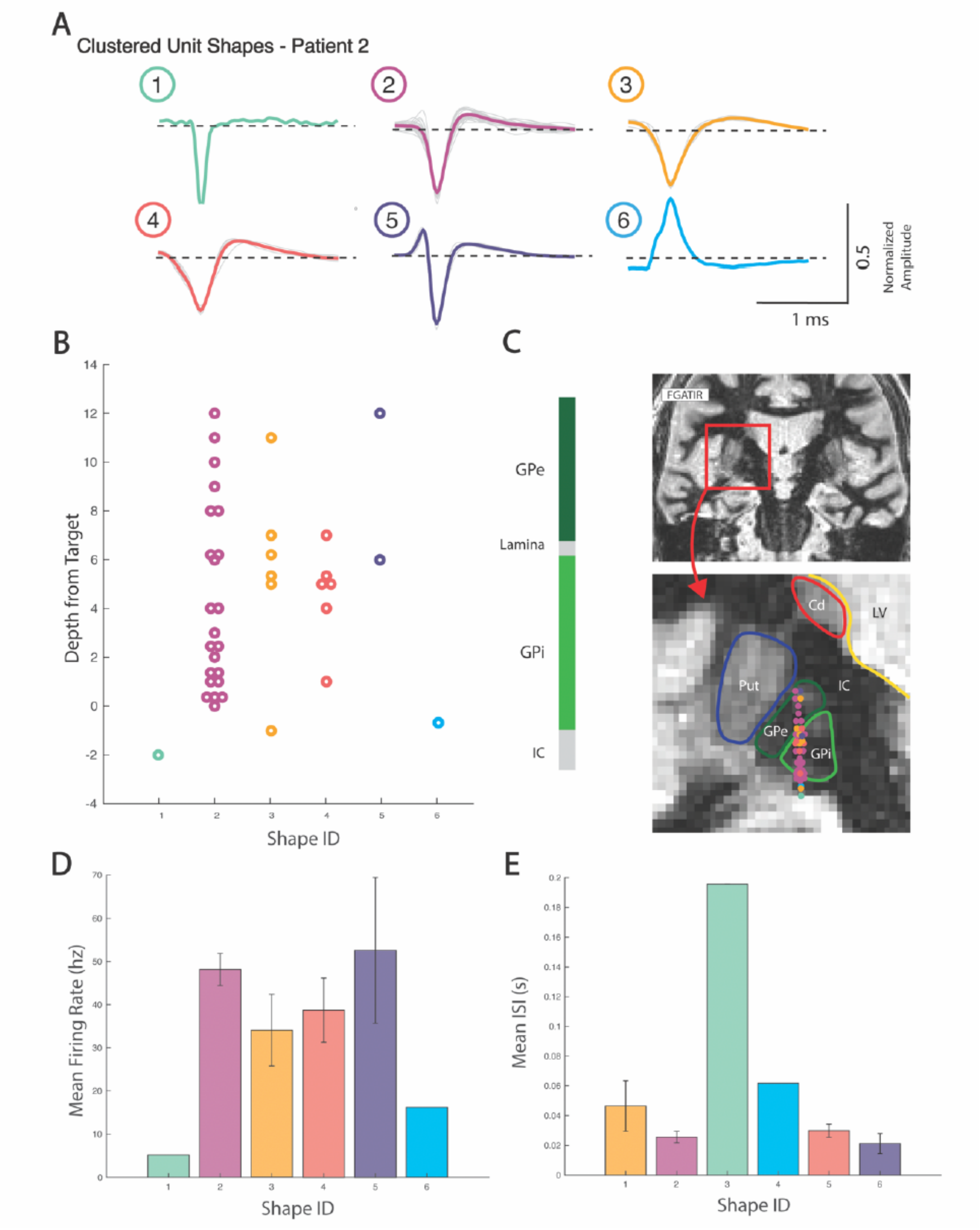

Figure S1 Caption:

Figure S1. Spike Shape Clustering for Patient 2. (A) Normalized unit waveforms and average cluster shape for the 6 unique shapes identified from hierarchical clustering. Shapes 2, 3, and 4 are the most common shapes, consistent with figures 7-8. **(B)** The 6 clustered spike shapes plotted by depth from target (mm) in the internal globus pallidus (GPi). **(C)** Spike shapes are

plotted along the visualized microelectrode recording track on hand-segmented anatomy on psuedocoronal FGATIR MRI. Average +/- SEM firing rate **(D)** and interspike interval **(E)** across shape cluster.

2. There is no limitations section in the paper, which should be added. For instance, the speculation about cell-type-specific waveforms is not based on any ground truth analysis. There is no histology or other method to prove this association, and it's certainly possible that other factors influence the recorded AP shape, such as the density of glial cells, the orientation of the cell, or neuromodulatory tone (from other projections). I still think the authors are definitely on to something, and this is a valuable analysis, but they should acknowledge that it's not foolproof.

The reviewer brings up a very important point that we are examining differences in spike shape morphology, which can be attributed to many different things beyond just cell-type differences and that ground truth confirmation would require a mixture of transgenic animal models, optogenetics, histology, etc. While we mention some of these things throughout the discussion, we agree it is important enough to add a dedicated limitations section (which we have done) and emphasize these points.

Limitations section excerpt:

"While we observed distinct spike shapes across anatomical regions that were largely consistent in both patients, other factors beyond cell-type such as cellular orientation, glial interactions, and neuromodulatory tone may influence waveform structure. While our method normalizes unit amplitude to reduce the impact of electrode distance, any ground truth assessment of cell-type would require a mixture of transgenic animal models, optogenetics, histology, and others."

3. Could the authors comment on how enriching these data with more contextual information could provide additional insights? For example, a more sophisticated model that includes the presumed anatomical region, the interspike interval of detected units, etc., may be able to constrain the classification of imputed neuron clusters.

We agree, and our ongoing future direction is attempting to create classifier models based on spike morphology features combined with firing properties and comparing it to expert segmentation from clinical imaging and MER analysis. We have more explicitly stated this as a part of the limitations section.

Limitations section excerpt:

"First, this study was conducted on densely sampled recordings from a small number of patients, which limits the generalizability of our findings. Future studies will assess group-level clustering and create classifier models to predict clinically-labeled boundaries from spike shapes and firing."

4. I may have missed this, but would it be possible, in principle, to reassess a recording with the identified putative neuron AP shapes as a means of improved spike detection—perhaps detecting smaller AP waveform amplitudes and enriching the number of detected units?

This is a very interesting idea, and would likely be possible to serve as another template-matching step to further improve current spike detection methods through something like a matching pursuit method. Rather than relying solely on amplitude-based thresholding, this approach could match putative spikes based on shape similarity. While our current analysis still relies on manual spike sorting, future work could explore how these principles could be incorporated into a more automated and sensitive method. We have added a sentence in the limitations section to acknowledge this possibility.

Updated Limitations Section:

“While this approach offers advantages over existing techniques, such as empirical spike windowing and shape based similarity, it is designed to compliment, not replace, standard spike sorting and shape comparison methods. Although, principles could be integrated into automated pipelines to enhance spike detection and unit classification similar to other matching pursuit methods.”

Citation added:

Lee JH, Carlson DE, Shokri Razaghi H, Yao W, Goetz GA, Hagen E, et al. YASS: yet another spike sorter. Advances in neural information processing systems. 2017;30.

5. The authors note that detected spikes can be regressed out of the ongoing time series to provide an estimate of the LFP. Could they comment on how the recording amplifier’s phase response (which is likely not flat across the frequency spectrum) may or may not lead to some distortions here?

We appreciate this important point, and acknowledge it is an inherent limitation in the analysis for figure 10. We have added this consideration in the results section.

Updated Results Section:

“For example, there is a clear decrease in spectral power above 150Hz when the spike shape contamination is removed. If the act of removing spikes introduced discontinuities (i.e. created artifactual contamination itself), this process of spike removal would instead result in an artificial increase in power. One important caveat, however, is that the phase response of the recording amplifier may not be flat across the frequency spectrum. This could introduce distortions into both the spike waveforms and the reconstructed residual signal, particularly in higher-frequency bands, and may limit the interpretability of subtle spectral features in this range.”

Reviewer #2: In this manuscript, Baker and colleagues introduce an approach for parameterizing the morphology of extracellular action potential recordings captured from the human brain. Briefly, the authors examine micro-electrode recordings of single units, and specifically the shape of the single units, in order to parameterize their structure and their duration of activity. They use this parameterization to hierarchically group spike shapes into clusters, and conclude by examining the relation between these clusters and anatomy. The main contribution here is that the authors use an approach built upon canonical response parameterization. In effect, this allows one to identify the precise duration of a single action potential so that the waveform between those time points can be compared to other action potentials in the recorded trace. By adopting this approach, for every single spiking event, the authors can extract out the specific duration of that spike, the scaling factor or amplitude (alpha) required to match that event to the template for that unit, and the residual LFP trace. This is an interesting approach, and could certainly be helpful for researchers focused on single unit spiking activity. However, there are some suggestions that could strengthen the overall conclusions of the work.

1. The study presented here appears to be largely a methodological report. How and why this could offer advantages over other approaches, however, is not clear. For example, if one were to simply group spiking events based on the template waveform extracted through spike sorting, would that yield results that are inferior to those presented here. In general, the paper presents an approach for extracting these features, but it is not clear how or why this approach should be better suited to human data than other approaches that are more routinely used in the literature.

We thank the reviewer for their thoughtful comments. We would like to clarify that the scope of our work is mainly a methodological addition, that adds a set of layers on top of standard clustering approaches involving spike sorting and unit parameterization. We begin with the spike sorted template, then identify a statistical layer of identification of when spike structure emerges and later disappears from the background signal. This parameterization then allows us to remove only the information maximally related

to the spike, allowing for more precise comparisons between shapes and preserved underlying structure of the residual LFP.

Simply grouping spikes (k-means, etc) based on extracted waveforms from spike sorting would be an alternative. However, statistically defined spike windows, amplitude-independent shape comparisons, and a more intuitive clustering approach of spike similarity (as opposed to a more abstract, unitless space) are key advantages built into our method. Although, as we note in the new limitations section, there is not a ground truth for cell-types to grade these different clustering methods. Future work on classification models of spike shape to predict clinical-determined anatomy could at least help determine which methods are most useful. To show how a more straightforward clustering method could affect spike shapes we used unsupervised 2D PCA on mean spike units from spike sorting and used K-means clustering (specified 6 clusters as in Figure 7), and compared the overlap in clustering assignment with our method using the Hungarian method, a combinatorial optimization algorithm. We show that amplitude normalization is a critical step and becomes a dominant feature in the clustering in Figure S2A (52% overlap). When waveforms are normalized prior to PCA, we observe shapes that are more in line with what we found, but still only shares 66% overlap with our method.

Figure S2:

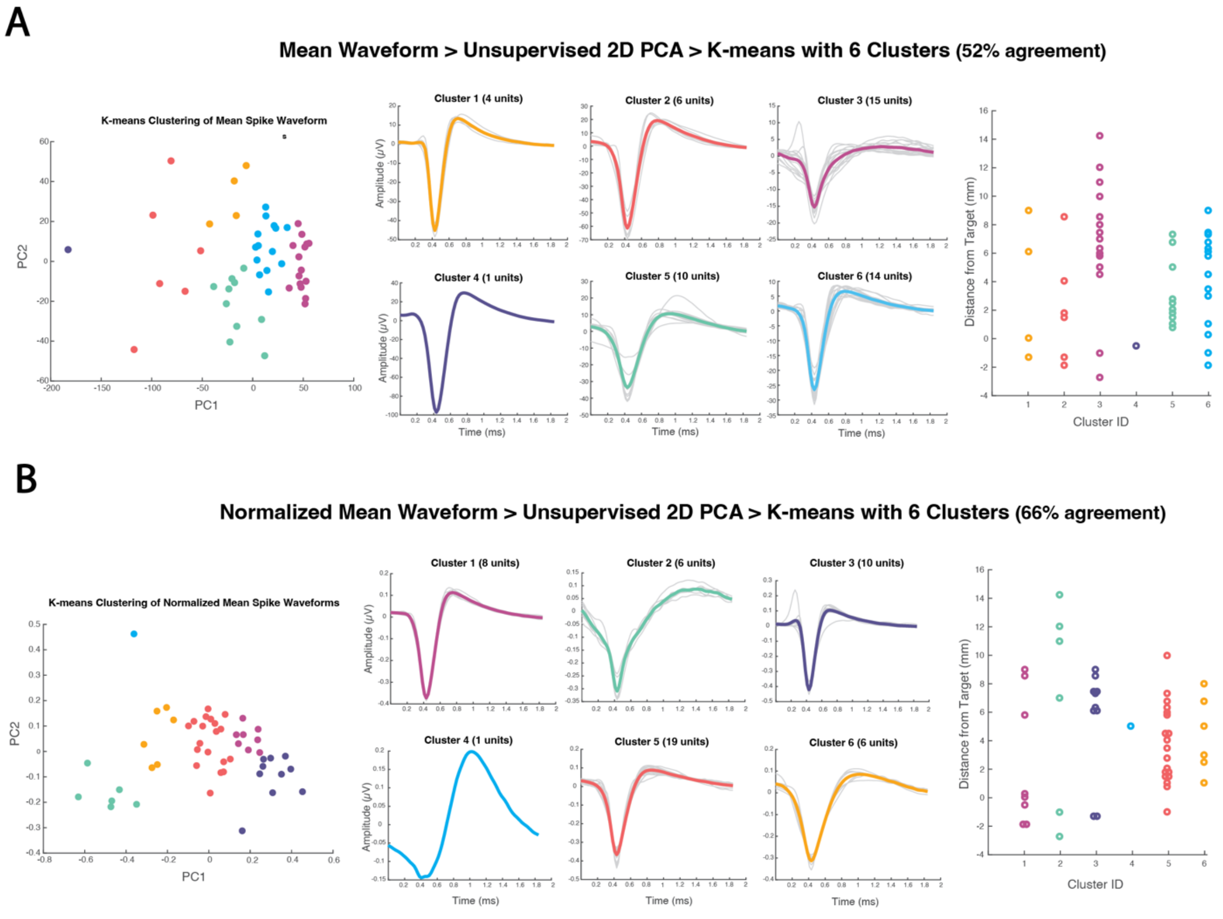

Figure S2 Caption:

“**Figure S2. Comparative Clustering Methods.** We compared hierarchical clustering outcomes from Figures 7-8 to directly clustering spike-sorted mean waveforms through unsupervised PCA (2D) followed by K-means clustering (specified same number of clusters)

and compared overlap using the Hungarian method, a combinatorial optimization algorithm. **(A)** Directly clustering mean unit waveforms shared 52% overlap with the hierarchical clustering method, and were heavily influenced by unit amplitude. **(B)** By first normalizing the mean unit waveforms prior to PCA and clustering, the spike shapes more closely resembled our clustering findings, but still only shared 66% overlap."

Added Results/Discussion Text:

"Comparison to Conventional Waveform Clustering Methods

To compare our shape-based clustering approach with more conventional methods, we performed an unsupervised analysis in which mean spike waveforms were clustered using principal component analysis (PCA) followed by k-means clustering. PCA was applied using the full waveform as a feature vector, and k-means clustering was performed with six specified clusters. The Hungarian method, a clustering optimization algorithm, was used to quantify cluster assignment overlap. When using unnormalized waveforms, clustering results were dominated by amplitude differences, and cluster morphology diverged substantially from those derived via our CRP-based similarity metric (Figure S2A; 52%). After normalizing waveforms to remove amplitude as a confound, clustering results more closely resembled our shape-based clusters; however, agreement remained partial (Figure S2B; 66%)."

Added Citation:

Kuhn HW. The Hungarian method for the assignment problem. Naval research logistics quarterly. 1955;2(1-2):83–97.

Updated Limitations Section:

"While this approach offers advantages over existing techniques, such as empirical spike windowing and shape based similarity, it is designed to compliment, not replace, standard spike sorting and shape comparison methods. Although, principles could be integrated into automated pipelines to enhance spike detection and unit classification similar to other matching pursuit methods."

2. One area where this could potentially be improved is in describing the results of this approach on neural data. However, here, the data and results presented are somewhat sparse. As far as I can tell, the hierarchical clustering result demonstrating the different anatomic correlates of different waveform clusters are all extracted from a single recording track in a single patient. The other main examples demonstrating how this approach can be helpful are correlating scaling factors with cardiac rhythms and extracting the residual LFP. These also appear to be single examples. The conclusions and the utility of this approach could be much improved if these analyses were applied across a larger set of subjects.

We agree that the current study is primarily methodological in scope and is intended as an initial demonstration of the approach's feasibility and potential utility. While many of the results are drawn from densely sampled recording sites of single patients, we see this as a foundation for future work applying these methods to large group-level data to create classifier models to further validate and expand the biological interpretations. Similarly in response to reviewer one's comment, we did add additional clustering analysis on spike shapes from the 3rd patient which showed the same 3 most common shapes of sorted units across similar spans. We also make these limitations clearer in a dedicated discussion section as noted in response to reviewer 1.

*See reviewer 1 #1 for full limitations section and updated supporting information figure.

Reviewer #3: Baker et al. present an intriguing spike sorting and parameterization method applicable to DBS MER. This technique potentially offers an objective characterization of a process traditionally reliant

on clinical expertise. However, several clarifications are necessary to fully interpret the study's results:

1. The authors mention obtaining recordings with the patient at rest. It is essential to clarify whether recordings involving somatosensory evoked potential testing or visual evoked potential testing were excluded. Additionally, were microstimulation or macrostimulation recordings included in the dataset?

Thank you for pointing this out. All recordings included in this study were pure baseline recordings, acquired while patients were at rest and without any evoked potential testing, movement tasks, or clinical stimulation (micro- or macro-stimulation) during the recording periods. We have clarified this in the Methods section. Future studies incorporating evoked activity may help extend the utility of this approach.

Updated Methods Text:

“No somatosensory or visual evoked potential or micro/macrostimultation testing were included in the analyzed data.”

2. Were there any adjustments to the final DBS trajectory post-MER, ensuring that the DBS lead localization accurately represents the initial MER trajectory?

The position of each MER recording site was corrected relative to the final DBS lead position as seen on postoperative imaging, with any adjustments made in depth or track position. Overall, we corrected for vertical moves to the DBS lead, but all analyzed MER sites were on the same track as final lead placement.

Relevant Methods Text:

“The site of each recording relative to the final lead position was known, enabling its location on the patient's MRI as follows: First, a postoperative CT scan was co-registered to preoperative MRI sequences using a normalized mutual information approach implemented in SPM12. Then, the lead artifacts were manually identified, and MRI sequences were resliced in plane with the lead artifact using custom Matlab code (Fig 1C-D)(Richner et al., 2020). The offset of each recording site from the final lead position was applied, and recording sites were plotted on T1-weighted, T2-weighted, and Fluid-attenuated Gradient echo with Adiabatic T1 Inversion Recovery (FGATIR) MRI sequences.”

3. Lastly, were any anesthetic or CNS sedating agents administered during these cases?

Patients may have had light dexmedetomidine sedation at the beginning of the procedure, but would be discontinued at least 15 minutes prior to the start of MER. No additional CNS- active agents were administered during the recordings. We added this clarification to the methods section. This could also be particularly interesting for group-level analyses to explore the consistency in spike shape across anesthesia states, which may be more consistent than firing properties.

Updated Methods Text:

“Patient's may have had light dexmedetomidine sedation at the beginning of the surgery, but would be discontinued at least 15 minutes prior to the start of MER. No additional CNS-active agents were administered during the recordings.”

4. Can the authors provide information on the background and expertise of the individual who conducted the manual segmentation of the basal ganglia?

MRB (PhD neurophysiologist) and KJM (MD neurosurgeon, PhD in neurobiology) hand-segmented visible basal ganglia boundaries. This has been added to the methods section.

Updated Methods Text:

MRI borders of the GPi and surrounding structures were visually identified and outlined in the figures by MRB and KJM (Fig 1E).

5. The study describes two GPi and one VIM case used to develop the technique. However, results are reported for only one GPi and one VIM case. Was the technique equally successful in the second GPi case?

Thank you for raising this important point. The third patient (second GPi case) was originally used for illustrative figures and representative traces. In response to reviewer feedback regarding generalizability, we have now included the full spike shape analysis for this second GPi patient as a new supplementary figure. Notably, we observed the same three major spike shape clusters, with similar anatomical spans across the globus pallidus, consistent with our findings from the first GPi case.

*see reviewer 1 #1 response for new supporting information figure and caption, and added limitation section.

6. The authors identified four distinct morphological populations within the white matter tracts surrounding the basal ganglia. Can they comment on the degree of heterogeneity suggested by this method? The Biological Interpretation section mentions an "apparent absence of identifiable unit activity" in these areas. Does this indicate true cells or noise? If it is noise, can this technique be used to identify it in an automated fashion?

The units observed at the very top and bottom of the MER track correspond to white matter regions and likely represent a mixture of background activity, noise, and weak or sparse spiking. We have clarified in the revised manuscript that these units may not reflect clean or canonical shaped single-units. As the reviewer insightfully suggests, even if some of this activity reflects noise, it may still serve a useful role in distinguishing white from gray matter based on the presence or absence of structured spike shapes—though the biological interpretation should be made with appropriate caution. We have included this clarification in the Discussion.

Updated Limitations Section:

“Additionally, some spike shapes observed in white matter regions may reflect some combination of background activity and amplifier/background noise rather than discrete units and should be interpreted cautiously. Regardless, even reliably identifiable shapes from background spiking/noise could still prove useful for classifier models.”

7. The use of the term "local field potential" should be clarified, as LFPs are traditionally considered slow propagations (less than 100 Hz). Would LFP isolation, as demonstrated in Figure 10, applied to the 1-100 Hz band range, also show notable separation?

We appreciate this important point. While LFP can sometimes be used to refer specifically to low-frequency oscillatory activity (<100 Hz), we use the term more broadly in this study to refer to the non-spiking component of the extracellular voltage signal. This includes both narrow-band low frequency oscillations as well as broadband activity (1/f shape of PSD), which reflects asynchronous population firing. In our analysis, spike removal primarily reduces high frequency power (>150 Hz), corresponding to

this broadband component, while no changes were observed in the lower 1-100 Hz range. We have clarified the broader usage of "LFP" in the manuscript to ensure clarity to the reader.

Updated Methods Text:

"Note, by LFP, we refer to an encompassing term for the non-spiking component of the extracellular voltage signal, which includes both low-frequency narrow-band oscillations and broadband activity (1/f shape of the PSD) which reflects asynchronous population firing".

8. In the 200-300 Hz range, does this represent the separation of spikes from ambient noise rather than LFPs?

The changes we observe in the high frequency range (>150Hz) with spike removal is not present in low frequency power where narrow-band oscillations are normally present. This likely reflects separation of spike-induced noise from broadband 1/f signal and ambient noise – not lower frequency narrow-band oscillations. As noted in #7,

---

Have the authors made all data and (if applicable) computational code underlying the findings in their manuscript fully available?
The PLOS Data policy requires authors to make all data and code underlying the findings described in their manuscript fully available without restriction, with rare exception (please refer to the Data Availability Statement in the manuscript PDF file). The data and code should be provided as part of the manuscript or its supporting information, or deposited to a public repository. For example, in addition to summary statistics, the data points behind means, medians and variance measures should be available. If there are restrictions on publicly sharing data or code —e.g. participant privacy or use of data from a third party—those must be specified.

Reviewer #1: Yes

Reviewer #2: None

Reviewer #3: None

PLOS authors have the option to publish the peer review history of their article (what does this mean?). If published, this will include your full peer review and any attached files.

If you choose "no", your identity will remain anonymous but your review may still be made public.

Do you want your identity to be public for this peer review? For information about this choice, including consent withdrawal, please see our Privacy Policy.

Reviewer #1: No

Reviewer #2: No

Reviewer #3: No
